# A chromosome-level genome assembly of an avivorous bat species (*Nyctalus aviator*)

**DOI:** 10.1038/s41597-024-03322-z

**Published:** 2024-05-10

**Authors:** Yang Geng, Yingying Liu, Yu Zhang, Lixin Gong, Yu Han, Zhenglanyi Huang, Can Ke, Hui Wu, Aiqing Lin, Jiang Feng, Tinglei Jiang

**Affiliations:** 1 Jilin Provincial Key Laboratory of Animal Resource Conservation and Utilization, Northeast Normal University, Changchun, 130117 China; 2 Key Laboratory of Molecular Epigenetics of Ministry of Education, School of Life Sciences, Northeast Normal University, Changchun, 130024 China; 3 College of Life Science, Jilin Agricultural University, Changchun, 130118 China; 4 Key Laboratory of Vegetation Ecology of Education Ministry, Institute of Grassland Science, Northeast Normal University, Changchun, 130024 China

**Keywords:** Comparative genomics, Genome

## Abstract

Currently, three carnivorous bat species, namely *Ia io*, *Nyctalus lasiopterus*, and *Nyctalus aviator*, are known to actively prey on seasonal migratory birds (hereinafter referred to as “avivorous bats”). However, the absence of reference genomes impedes a thorough comprehension of the molecular adaptations of avivorous bat species. Herein, we present the high-quality chromosome-scale reference genome of *N. aviator* based on PacBio subreads, DNBSEQ short-reads and Hi-C sequencing data. The genome assembly size of *N. aviator* is 1.77 Gb, with a scaffold N50 of 102 Mb, of which 99.8% assembly was anchored into 21 pseudo-chromosomes. After masking 635.1 Mb repetitive sequences, a total of 19,412 protein-coding genes were identified, of which 99.3% were functionally annotated. The genome assembly and gene prediction reached 96.1% and 96.1% completeness of Benchmarking Universal Single-Copy Orthologs (BUSCO), respectively. This chromosome-level reference genome of *N. aviator* fills a gap in the existing information on the genomes of carnivorous bats, especially avivorous ones, and will be valuable for mechanism of adaptations to dietary niche expansion in bat species.

## Background & Summary

As an important component of the ecological niche, the dietary niche of animals reflects variations in their food intake, which influences their survival and reproduction^1^. Changes to the diet of animals may induce phenotypic variations to open new ecological opportunities^2^, such as physiological (i.e., nutrient assimilation and energy metabolism), morphological, and behavioral variations^3^. Consequently, studies on the genomic adaptations of species with dietary niche variations (i.e., niche expansion) could provide insight into the genetic mechanisms responsible for the ecological niche breadth evolution. Chiroptera (bat) species, serve as an excellent subjects for studying the evolutionary mechanisms of dietary niches due to their diverse diets, which include insectivory, carnivory, piscivory, frugivory, nectarivory, and sanguivory^4^.

Currently, three carnivorous bat species, namely *Ia io*, *Nyctalus lasiopterus*, and *Nyctalus aviator*, are known to actively prey on seasonal migratory birds^5–7^. They usually consume insects in summer and prey on nocturnal migratory birds through an aerial-hawking strategy during spring and autumn. Comparing to closely related insectivorous bat species, the dietary niches of avivorous bat species have expanded from insects to birds^8,9^. Previous studies have identified similarities in the morphology and behavior of three avivorous bat species. However, there remains a lack of understanding of the molecular mechanisms that drive the evolution of this specific feeding habit. For example, previous research has identified physiological adaptations related to avivorous diet by comparing the genomes of *I. io* against other bat species^8^. However, it remains unknown whether these adaptations are also present in *N. aviator* and *N. lasiopterus*, which are distantly related. Additionally, the direct interactions between avivorous bat species and birds contribute to the transmission of viruses^10,11^. For instance, the typical influenza A virus (IAV) is capable of infecting bat cells, and H9 IAV has been identified in bats^12^. Recently, the hemaglutinin (HA) gene of H19 IAV, which was isolated from a wild duck, has exhibited characteristics of both avian and bat influenza viruses^13^. However, little is known about the adaptation of immunity in avivorous bat species. These issues merit further investigation to achieve a more comprehensive understanding of the genetic basis underlying the dietary niche evolution, particularly in distantly related taxa with similar expansion in dietary niche. Nonetheless, the absence of genomes of high quality impedes the possibility of conducting comprehensive research.

**Fig. 1:**
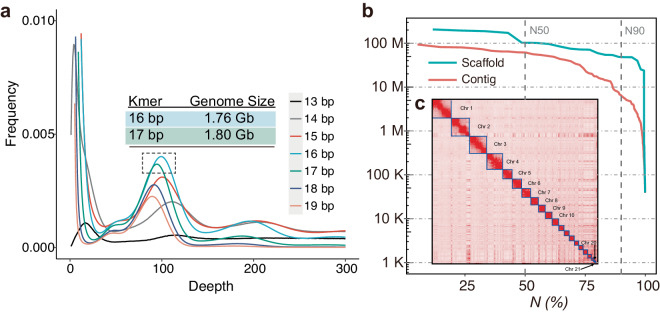
The results of genome assembly for *N. aviator*. (**a**) Genome size estimation by different kmers. The estimated genome size of *N. aviator*, based on 16 bp and 17 bp kmers, produced consistent outcomes, suggesting a genome size of around 1.8 Gb. (**b**) Length distribution of genome assembly at contig- (red) and scaffold-level (green). It indicates the percentage (x%) of the assembly that consists of contigs and scaffolds of at least a certain size. (**c**) Hi-C Map for *N. aviator*. The chromosomes have been ordered by size.

**Fig. 2:**
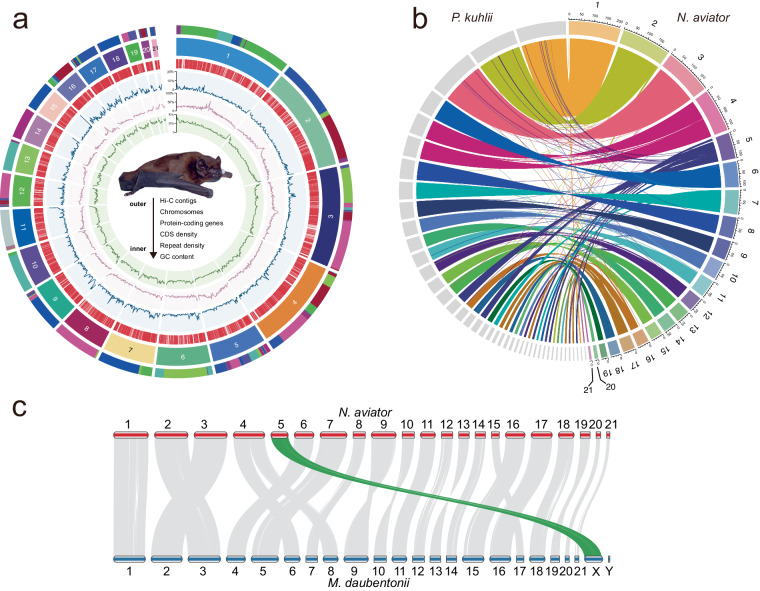
The circos plots depict the genomic structure and genome syntenic blocks of *N. aviator*. (**a**) The tracks, arranged from outer to inner, represent the contigs that make up the scaffolds (adjacent contigs are shown in different colors), 21 chromosome-level scaffolds (The scaffolds were sorted by length, with 1 representing the longest and 21 the shortest), positions of protein-coding genes, density of CDS, density of repetitive sequences, and GC content. CDS density, repetitive sequence density, and GC content are calculated based on 1 Mb windows. (**b**) The circos plot illustrates the syntenic blocks shared between *N. aviator* and *P. kuhlii*. Only scaffolds over 4 Mb and syntenic blocks larger than 5 kb are depicted. (**c**) The pairwise synteny among *N. aviator* and *M. daubentonii*. The identified X chromosome of *N. aviator* was highlighted in green.

**Fig. 3:**
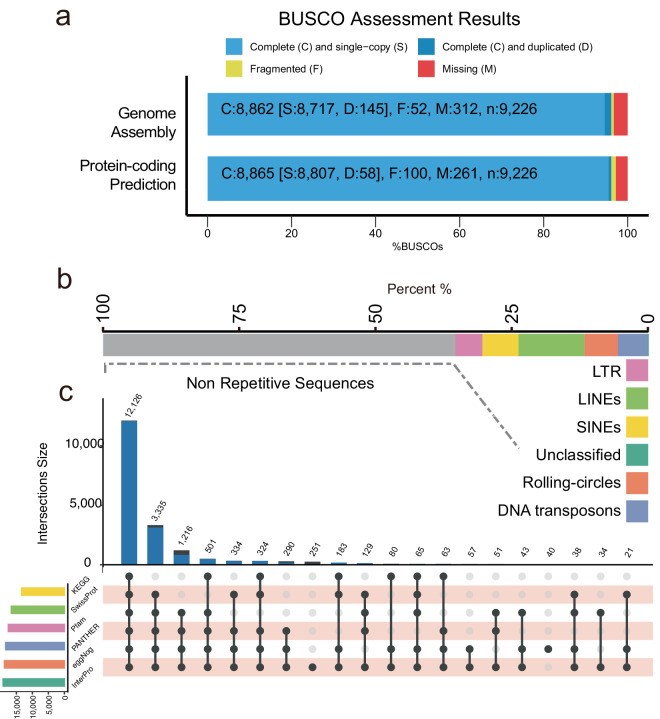
The functional annotation and completeness of the genome of *N. aviator*. (**a**) The genome assembly and protein-coding gene prediction were assessed using BUSCO. Both the completeness of the assembly and the prediction are over 96%, indicative of high quality. (**b**) The stacked histogram depicts the proportion of repeated sequences in the genome assembly. Each color represents a different type of repetitive sequence. (**c**) The upset plot displays the functional annotation of predicted protein-coding genes.

Here, we presented a high-quality chromosome-level genome assembly of *N. aviator* using a combination of PacBio subreads (299.16 Gb), DNBSEQ short reads (200.25 Gb), and high-throughput chromatin conformation capture (Hi-C) sequencing data (200.17 Gb) (Table 1). The genome survey revealed an estimated genome size of approximately 1.8 Gb for *N. aviator* Fig. 1a. Finally, we generate a 1.77 Gb genome assembly of *N. aviator* with contig N50 and scaffold N50 of 61.24 Mb and 101.86 Mb, respectively (Fig. 1b, Table 2). Approximately 99.8% of genome sequences were mounted to 21 chromosome-level (20 autosomes and X chromosome) scaffolds (Figs. 1c, 2a), which is consistent with the diploid chromosome number of *N. aviator* (2n = 42)^14^. The whole-genome synteny analysis showed a strong synteny (>93%) among *N. aviator* and closely related species *Pipistrellus kuhlii* (GCF_014108245.1^15^, Fig. 2b). The synteny analysis using the chromosome-level genome of *Myotis daubentonii* (GCF_963259705.1^16^) revealed that the genome assembly of *N. aviator* has attained chromosome-level resolution and successfully characterized the X chromosome (Fig. 2c). The genome assembly consisted of 635.1 Mb (35.75%) repetitive sequences (Fig. 3a, Table 3). After masking repetitive sequences, a total of 19,412 protein-coding genes were predicted, and 99.07% of them were functionally annotated (Fig. 3b, Tables 4, 5). The assessment using Benchmarking Universal Single-Copy Orthologs (BUSCO) revealed 96.1% completion rates for both genome assembly and annotation, as shown in Fig. 3c. This indicating a high-quality assembly and annotation of the genome. In summary, the genome assembly of *N. aviator* establishes a foundation for comprehending the genetic adaptation of bat species with diverse diets and serves as a valuable resource for conducting further studies on the evolutionary mechanisms of dietary niche expansion.

**Table 1:** Statistics on the genome sequencing data of *N. aviator*.

Library	Reads number	data (Gb)	Max length (bp)
PacBio	15,539,399	298.86	368,564
DNBSEQ	667,487,037	200.25	/
Hi-C	667,262,960	200.18	/

**Table 2:** Statistics on genome assembly and the genome evaluation for *N. aviator*.

Genome assembly statistics	
size (bp)	1,776,615,751
contig number	337
contig N50	61 Mb
scaffold number	195
scaffold N50	101 Mb
GC content (%)	42.7
Hi-C loading rate (%)	99.45
Percent gaps (%)	0.002
**Genome assembly consistency**	
PacBio subreads mapping rate (%)	99.96
DNBSEQ short reads mapping rate (%)	99.29
DNASEQ short reads coverage > 4X (%)	99.71
DNASEQ short reads coverage > 10X (%)	99.55
DNASEQ short reads coverage > 20X (%)	99.27
DNASEQ short reads coverage > 40X (%)	98.23
**BUSCO completeness of genome assembly (a total of 9,926 orthologes)**	
Complete BUSCOs	8,862
Complete and single-copy BUSCOs	8,717
Complete and duplicated BUSCOs	145
Fragmented BUSCOs	52
Missing BUSCOs	312

**Table 3:** Summary of repetitive elements in the genome assembly of *N. aviator*.

Repeat Classes	Count	Length (bp)	Percent (%)
DNA transposons	804,159	96,517,425	5.43
SINEs	637,769	117,935,009	6.64
LINEs	496,985	215,002,783	12.1
LTR elements	377,645	87,159,734	4.91
Total interspersed repeats		520,421,438	29.29
Rolling-cicles	644,248	108,683,329	6.12
Unclassified	27,474	3,806,487	0.21
Satellites	17,899	1,497,615	0.08
Simple repeats	43,125	5,687,426	0.32
Low complexity	1,814	327,949	0.02
Total masked		635,129,906	35.75

**Table 4:** Statistics on protein-coding genes prediction with three strategies for *N. aviator*, ‘*’ indicate that the final dataset excluded potential non-coding and short coding genes (coding protein sequence < 50 aa), while preserving the longest transcript for each gene.

Gene set	gene number	transcript number	Average transcript length(bp)	Average CDS length(bp)	Average exons per gene	Average exon length(bp)
Braker3	38,290	44,555	38,006.71	171.79	8.97	171.70
miniprot	/	58,941	48,326.22	166.43	/	/
TOGA	53,428	53,428	31,077.71	165	8.57	165
StringTie	/	61,318	36,801.89	/	8.37	322.73
TransDecoder	20,742	57,593	46,048.91	169.73	29.51	337.68
PASA	/	315,642	8,064.39	/	2.71	409.34
EVM	38,879	38,879	22,535.98	182.2	5.97	182.2
PASA-update	38,812	50,258	28,936.69	173.44	9.96	273.78
Final set*	19,412	19,412	36,782.05	174.47	9.74	259.47

‘/’ indicate that the software is unable to predict the corresponding evidence.

**Table 5:** Summary of gene functional annotation of genome assembly for *N. aviator*.

Database	Number	Percent (%)
eggNOG	18,769	96.69
SwissProt	16,607	85.55
InterPro	19,188	98.52
All	19,277	99.30

## Methods

### Sample collection and sequencing

In this research, a female healthy *N. aviator* individual was randomly captured on September 15, 2021, in Congjiang county, Xingyi city, Guizhou province, China. This individual was anesthetized with ether prior to the euthanasia procedure (cervical dislocation). The nine tissues (muscle, brain, lung, liver, heart, ovary, spleen, kidney, and stomach) were sampled for DNA and RNA extraction. All tissues were frozen immediately using liquid nitrogen and then were stored in a −80 °C freezer. For genome sequencing, genomic DNA was extracted from muscle tissue for three sequencing libraries construction, PacBio CLR library (20–40 kb), DNBSEQ library (paried-end 150 bp), and Hi-C library (paried-end 150 bp). The subreads were sequenced using the PacBio Sequel II platform, the short reads and the Hi-C reads were sequenced using DNBSEQ platform. The raw data of DNBSEQ short reads and Hi-C sequencing data were filtered using SOAPnuke version 2.1.5^17^. For transcriptome sequencing, total RNA was extracted from each tissue and used for constructing sequencing libraries (paired-end 150 bp), respectively. A total of nine libraries were sequenced on BGISEQ platform. All transcriptome sequencing data was also filtered using SOAPnuke. To ensure the quality of sequencing data, all fastq files were filtered using fastp version 0.23.4 (with parameter: -q 20)^18^ to exclude sequences with a phread score below 20, except for PacBio subreads. All procedures involving the capture of bats and experimental procedures were approved by the Science and Technology Ethics Committee of Northeast Normal University, China (permit ID: NENU-202302001).

### Genome assembly

#### Genome survey

The GCE (genomic charactor estimator) version 1.02^19^ was used to assess the genome size of *N. aviator* based on 200.25 Gb clean short reads before genome assembly. A range of kmers (13–19 bp) lengths were used to estimated genome size of *N. aviator*. The genome size of *N. aviator* was estimated to be approximately 1.8 Gb based on the assessment results when using kmer lengths of both 16 and 17 bp. The subsequent assembly of the genome was guided by the genome size of 1.8 Gb (Fig. 1a).

#### Genome assembly

A total of 299.16 Gb PacBio subreads were corrected using NextDenovo version 2.5.0 (https://github.com/Nextomics/NextDenovo). Subsequently, the corrected subreads were pairwise aligned with each other using kbm2 (with parameters: -t 10 -c 2) from the WTDBG2 version 2.5^20^. Several rounds of parameter optimization (with parameters: -A --node-drop 0.25 --node-len [1536, 2048, 2304, 2560] --node-max 400 -s [0.05, 0.07] -e 3 --rescue-low-cov-edges --no-read-length-sort --aln-dovetail [4608, 9216, -1]) were conducted to attain optimal assembly results. The results of parameter optimizations were sorted based on the contig N50 of the assembly, and the longest one was retained. The consensus sequence of the best assembly result is obtained by using wtpoa-cns from WTDBG2. NextPolish version 1.4.0^21^ was utilized to correct the assembly results of WTDBG2, aiming to reduce the assembly error rate. Both PacBio subreads and DNBSEQ short reads were employed for this correction. The error-corrected results served as the final contig-level assembly of *N. aviator* for subsequent analysis.

#### Hi-C scaffolding

The Hi-C sequencing data was employed to extend the contig-level assembly, expanding the contig into a chromosome-level scaffold. A total of 200.17 Gb Hi-C sequencing data was filtered using HiC-Pro version 3.1.0^22^. The filtered valid pairs were aligned to the contig assembly using chromap version 0.2.4-r467^23^. Subsequently, the chromosome-level scaffold assembly was performed using YaHS version 1.2a.1^24^. The scaffold assembly was visualized using Juicebox Assembly Tools version 2.20^25^ and corrected manually. The final assembly was generated using YaHS based on the reviewed assembly file mentioned above. The completeness of the genome assembly was assessed using BUSCO version 5.2.2^26^ with the mammal database (with mammalia_odb10).

### Genome annotation

The EDTA version 2.0.0 (with parameters: --sensitive 1 --anno 1 --evaluate 1) was used to annotate repeat elements of *N. aviator* genome assembly^27^. The CDS sequences of *P. kuhlii* were used as input of EDTA to improve the accuracy. The high-quality repeat sequence data of six bats described in this article^28^ was download and used as curated library in EDTA. The genomic region containing repetitive sequences was masked and utilized for subsequent analyses. The protein-coding genes were predicted with three different strategies: (1) de novo prediction; (2) homology-based prediction; 3) transcriptome-based prediction. All cleaned transcriptome sequences of all tissues of *N. aviator* were mapped to genome using HISAT2 version 2.2.1^29^ and were assembled using StringTie version 2.2.1^30^. The transcriptome assembly identified coding regions by utilizing TransDecoder version 5.5.0 (https://github.com/TransDecoder/TransDecoder) and constructing the transcriptome database with PASA version 2.5.2^31^. For homology-based prediction, the proteins sequences of bat species were extracted from OrthoDB11^32^, and alignment against the genome assembly of *N. aviator* using miniprot version 0.11^33^. For de novo prediction, the protein sequences and transcriptome alignments mentioned above were used to generate gene prediction by using Braker3 version 3.0.6^34^. In order to enhance the annotation results, we utilized the transcriptome evidence classified as ‘I’, ‘PI’, and ‘UL’ by TOGA version 1.1.6^35^ as addition evidence. With humans genome as a reference, the genome assembly of *N. aviator* was aligned to reference by using make_lastz_chain (https://github.com/hillerlab/make_lastz_chains) to create a pairwise genome alignment, serving as input for TOGA. The evidence of gene prediction mentioned above was integrated by EvidenceModeler (referred to as EVM in Table 4) version 2.1.1^36^ with (1) evidence of Braker3 set to weight 1; (2) the evidence of miniprot set to weight 3; (3) the evidence of PASA set to weight 10; 4) the evidence of TransDecoder and TOGA set to weight 8. Then, two rounds of PASA were conducted to update the integrated gene predictions. We extracted protein-coding sequences from annotation results, and translated them into protein. The short protein sequences ( < 50 aa) were removed. Filtered annotation results were aligned to proteins of mammalian database of RefSeq non-redundant protein sequence database (referred to as NR in Table 5) using DIAMOND version 2.0.14^37^. Potential noncoding (e-value of hits < 1e-5) sequences were removed. In total, 19,412 protein-coding genes were predicted in *N. aviator* genome with an average transcript and coding sequences (CDS) length of 36,782.05 bp and 174.47 bp, respectively (Table 4). The proteins coded by genes were search against the SwissProt mammalian database using DIAMOND version 2.0.14 (with parameter: blastp -e 1e-5), the eggNOG database using eggNOG-mapper version 2.1.7^38^, and InterPro database using InterProScan version 5.65-97.0^39^ (Table 5).

### Identification of X chromosome

Based on the karyological studies of *N. aviator*, the karyotype of *Nyctalus* species is strikingly similar to that of *Myotis* species. We select *M. daubentonii* as reference. The protein-coding genes and annotations of *M. daubentonii* were download from NCBI RedSeq database (accession: GCF_963259705.1^16^) whose X chromosome had been identified. The MCscan (python version)^40^ was used to identity synteny between *M. daubentonii* and *N. aviator*. The X chromosome of *N. aviator* was identified based on the syntenic blocks.

## Data Records

The final genome assembly of *N. aviator* has been submitted to the GeneBank database under the accession number GCA_036971965.1^41^ and the Genome Warehouse in National Genomics Data Center under accession number GWHESEW00000000. The raw genome (PacBio, DNBSEQ short reads, Hi-C) and transcriptome sequencing data have been submitted to the Sequence Read Archive at NCBI under accession numbers SRP485754^42^.

## Technical Validation

The mapping rates of DNBSEQ short reads and PacBio subreads were 99.26% and 99.96%, respectively, of which, over 98% of the genome assembly with >40 × coverage. This suggests a significant level of consistency in the assembly of the genome. We employed the genome of *P. kuhlii* (GCF_014108245.1^15^) as a reference genome and utilized lastz version 1.04.00^43^ to align the genome of the *N. aviator* against the reference genome. Genome synteny analysis of *N. aviator* and *P. kuhlii* revealed that more than 93% of the genome assembly consists of syntenic blocks. The X chromosome of *N. aviator* was also successfully identified through pairwise synteny analysis between *N. aviator* and *M. daubentonii*. The BUSCO assessment revealed that the genome assembly of *N. aviator* contained 96.1% of orthologs from the mammalia_odb10 dataset, comprising 8717 single-copy, 145 duplicated, 52 fragmented, and 312 missing BUSCOs. Furthermore, the final gene annotation of the assembly annotated 96.1% of the orthologs from BUSCO, consisting of 8807 single-copy, 28 duplicated, 100 fragmented, and 261 missing BUSCOs.

## Data Availability

In this study, all analyses were conducted following the manuals and tutorials of software and pipeline. The detailed software versions are specified in the methods section. Unless specified otherwise, default or author-recommended parameters were used for software and analysis pipeline. Detailed information about the parameters and custom scripts utilized in this research can be obtained by downloading them from https://github.com/life404/genome-NycAvi.git.
